# Strong Solar Radiation Forces from Anomalously Reflecting Metasurfaces for Solar Sail Attitude Control

**DOI:** 10.1038/s41598-018-28133-2

**Published:** 2018-07-03

**Authors:** Dylan C. Ullery, Sina Soleymani, Andrew Heaton, Juan Orphee, Les Johnson, Rohan Sood, Patrick Kung, Seongsin M. Kim

**Affiliations:** 1 Electrical and Computer Engineering, University of Alabama, Tuscaloosa, 35487 United States; 2 NASA, Marshall Space Flight Center, Huntsville, 35811 United States; 3 Aerospace Engineering, University of Alabama, Tuscaloosa, 35487 United States

## Abstract

We examine the theoretical implications of incorporating metasurfaces on solar sails, and the effect they can have on the forces applied to the sail. This would enable a significant enhancement over state-of-the- art attitude control by demonstrating a novel, propellant-free and low-mass approach to induce a roll torque on the sail, which is a current limitation in present state-of-the-art technology. We do so by utilizing anomalous optical reflections from the metasurfaces to generate a net in-plane lateral force, which can lead to a net torque along the roll axis of the sail, in addition to the other spatial movements exhibited by the sail from solar radiation pressure. We characterize this net lateral force as a function of incidence angle. In addition, the influence of the phase gradients and anomalous conversion efficiencies characteristics of the metasurfaces are independently considered. The optimum incidence angle that corresponded with the maximum net lateral-to-normal force ratio was found to be −30° for a metasurface exhibiting 75% anomalous conversion efficiency with a phase gradient of 0:71*k*_0_.

## Introduction

Solar sails are large, flexible, reflective surfaces that provide propellant-free space travel from the momentum imparted upon them by photons from the Sun. Instead of requiring large supplies of chemical propellant stored onboard the spacecraft, a solar sail’s in-space propulsion is achieved from solar radiation pressure. Therefore, solar sails allow for a spacecraft to be lighter and have greater longevity. While the force density from this radiation pressure is small, for an object with a large enough area, significant thrust can be achieved. Since there is little resistance in space, this allows for the possibility of Sun-propelled spaceflight, even in deep space.

A pioneering example that paved the way for future development of solar sail research was the Interplanetary Kite-craft Accelerated by Radiation Of the Sun (IKAROS), made by the Japanese Aerospace Exploration Agency (JAXA). Launched in 2010, IKAROS was the first demonstration of a spacecraft controlled via solar sails. Attitude control was accomplished using liquid crystals along the surface of the sail. The success of IKAROS sparked further investigation into solar sail technology, leading to missions such as the Nano-Sail D mission, the LightSail projects, and the upcoming Near Earth Asteroid (NEA) Scout mission^1–8^.

Despite the allure of the sustainable, propellant-free option of solar sails, there are a few drawbacks that need to be addressed, primarily with regards to attitude control. There are already some attitude control methods available, though they typically involve onboard mechanical actuators^9^ that add mass and complexity to the system. Additionally, the current hardware often employs moving parts, which serves to further complicate the system and increase the likelihood of malfunction. In order to utilize solar sail technology to its full potential, a low mass alternative method to attitude control would be ideal. Furthermore, current solar sail attitude control systems do not effectively control movement about the roll axis (the axis normal to the sail surface). An alternative method for solar sail attitude control could be to utilize metasurfaces.

Metamaterials are sub-wavelength structures that are able to interact intimately with electromagnetic waves and manipulate their propagation by virtue of their geometry and arrangements. Metamaterials are captivating devices with optical properties that are not present in materials available in nature. The unique properties of these metamaterials can be attributed to their effective electrical permittivity and their magnetic permeability, which can be tuned, allowing for a variety of interesting effects^10^.

One phenomena exhibited by metamaterial surfaces that is of particular interest, is anomalous reflection of light. Typically, specularly reflected light will be reflected from a surface at the same angle to the normal as its original angle of incidence. With anomalously reflected light, however, the angle of reflection is altered, allowing light to be reflected at a wide variety of angles, even parallel to the metamaterial surface. The fact that the incident and anomalously reflected waves are no longer symmetric to each other with respect to the normal axis of the surface means that there will be a net tangential force. Also, the further the metasurfaces are from the center of the sail, the larger their contributions will be to the torque along the roll axis.

Metasurfaces are employed to reflect electromagnetic waves anomalously^11–13^. Anomalous reflection of light could be achieved by generating a periodic phase gradient, which could be designed by means of gradually changing the dimensions of plasmonic antennas or resonator arrays inside of each unit-cell of the metasurface^14–16^. The manipulation of the size and shape of each plasmonic antenna, and generally the dimensions of unit-cell, could provide us with specific frequency bands of operation in which we can observe irregular and engineered reflections^17^. Metasurfaces can be sensitive to specific polarizations of electromagnetic waves that rely on the methodology of their design or the shape and orientation of each plasmonic subunit^13,14,18,19^. While researchers examine various ideas to provide novel metasurfaces with better control over the reflection angle, they also try to improve the efficiency and effectiveness of these structures, which could be defined as the ratio of the reflected power of light in the desired angle to the total incident light power^20,21^. With this in regard, the efficiency factor of metasurfaces is also included in our calculations and it is discussed extensively. Additionally, there are several reports of controllable reflectances of the metasurfaces, which is getting more attention in recent years^21^.

The main motivation of this paper is to develop the founding analytical equations to describe the tangential forces on a solar sail from anomalously reflected light. Once the force vectors are calculated, the torque along each axes can be found by τ = **r** × **F**. To our knowledge, anomalous reflections from metasurfaces have not yet been considered for application to solar sail attitude control. Finding the expressions for the forces on the solar sail due to anomalously reflecting metasurfaces will provide fundamental engineering insight into how such metasurfaces can be an effective method of attitude control.

## Metasurface Devices

**Figure 1:**
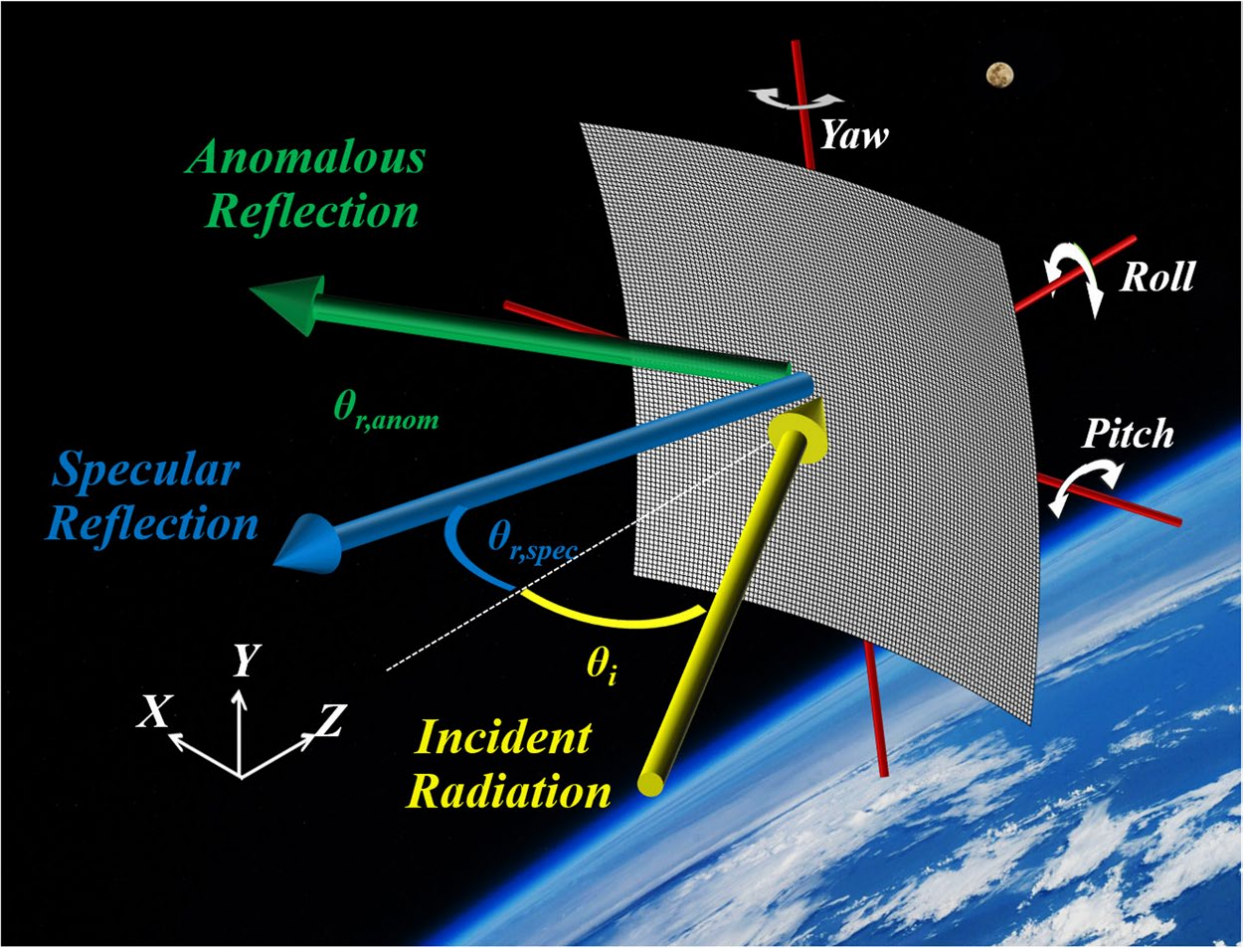
Schematic of solar sail system interacting with oncoming TE polarized electromagnetic field. Angle of incidence is varied along the x-z plane. Background image was provided by NASA.

**Figure 2:**
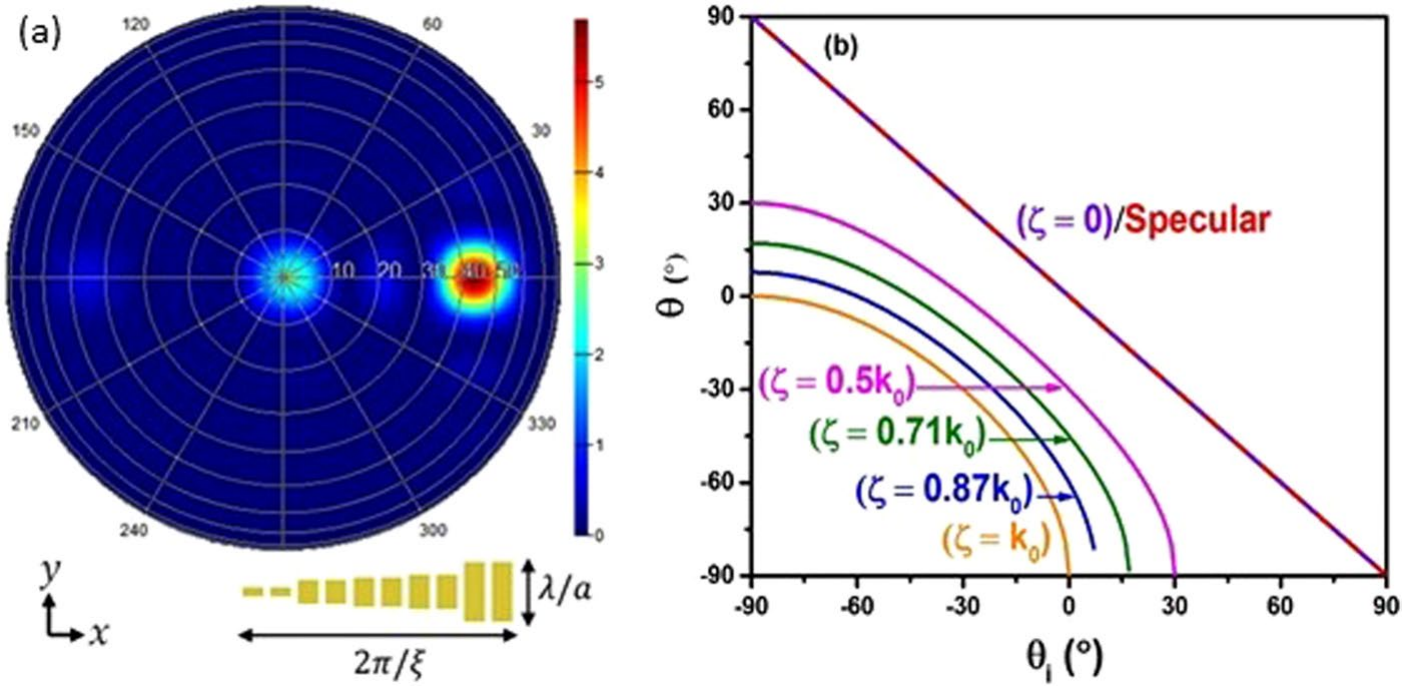
(**a**) Is a far field projection of the reflected electromagnetic wave with *θ*_*r*_ = 45°. Here *θ*_*i*_ is equal to 0° (i.e. normal to metasurface) and the polarization of electric field is in the $\widehat{y}$ direction. $\frac{\lambda }{a}$ and $\frac{2\pi }{\zeta }$ are the lattice constants for one unit-cell of the resonator arrays in $\widehat{y}$ and in $\widehat{x}$ directions, respectively. (**b**) Is a plot of different angles of reflections that arise from using generalized Snell’s law for anomalously reflected light. At *ζ* = 0, the angle of reflection is the same as specularly reflected light. In the plot *θ*_*r*,*spec*_ refers to the specularly reflected wave, which is opposite sign to the incidence angle *θ*_*r*,*anom*_ refers to the angles of reflection of the anomalously reflected waves.

Figure 1 illustrates a solar sail in space which moves due to a generated radiation power from the photon flux. Attitude control is typically regarded with respect to rotation about three principle axes centered upon the solar sail. As can be seen on the illustration in Fig. 1, pitch is the rotation about our chosen x-axis, yaw corresponds to rotation about the y-axis and roll corresponds to rotation about the z-axis. Here we consider a metasurface to be integrated on the surface of a solar sail that would activate the desired anomalous reflections in addition to the typical specular reflections. Our metasurface proposed here consist of a subwavelength nano-resonator array, and has a linear phase gradient surface for each unit. The unit-cell of the focused metasurface is presented in Fig. 2a. This figure demonstrates the far field projection of the reflected electromagnetic wave. It is apparent that the incident transverse electric (TE) (electric field polarized perpendicular to direction of propagation) wave at normal incidence to the metasurface is reflected anomalously at a 45° angle. The electric field polarization is in the y direction, which excites the plasmonic mode of each resonator. The coupling of the plasmonic modes provides us with the unusual reflection behavior. The results revealed that the efficiency of anomalously reflected light was more than 75%. This structure is simulated using the finite-difference time-domain (FDTD) method under a periodic boundary condition^14,22^.

The unit-cell of our metasurface consists of 10 gold patch nano-antennas with their longitudinal lengths, which is in the y-direction, gradually changing in the phase gradient direction (x-direction), and varying between 240 *nm* to 40 *nm* as it is presented in Fig. 2. The longitudinal plasmonic mode of each nano-antenna can be excited via TE polarized electromagnetic waves (in y-direction). As anticipated, each nano-antenna has a plasmonic resonance frequency in the visible light frequency band, which exhibits higher values upon reduction of the longitudinal dimensions of the antenna. The total dimensions of the phase gradient unit, or the unit-cell, of our metasurface, in the phase gradient direction (x-direction), has a determinative impact on the operational frequency band of the metasurface. According to the generalized Snell’s law, the phase gradient is equal to $\nabla {{\rm{\Phi }}}_{x}=\frac{{\lambda }_{0}}{{P}_{x}}$, where the *P*_*x*_ is the periodicity of the unit-cell in the x-direction. For instance, we assumed *P*_*x*_ = 1200 *nm*, so the cut-off wavelength of our metasurface is *λ*_0_ = 1200 *nm*.

## Methods

The forces from a plane wave on a solar sail can be found from using the Maxwell stress tensor in the law of conservation of momentum equation^23–25^. The incident wave, the reflected wave, and the transmitted wave, all have momentum and energy, so they will all generate a force on the solar sail^23,24^. In most cases, however, solar sails are made out of highly reflective materials that allow for no transmitted waves, so the transmitted term can be neglected. For convenience, the symbols and parameters used throughout this paper are summarized in Supplemental Table S1 in the online Supplementary Information.

The law of conservation of momentum is^23,24,26–31^

1 $${\bf{f}}+\varepsilon \mu \frac{\partial {\bf{S}}}{\partial t}=\nabla \cdot \overleftrightarrow{T},$$

where **f** is the force density. $\overleftrightarrow{T}$ is the Maxwell stress tensor, and it relates the momentum from electromagnetic fields to the momentum from a mechanical system, such as the solar sail. Since we are only observing the forces at a specific instant in time, $\frac{\partial {\bf{S}}}{\partial t}$ will be zero. The Maxwell stress tensor is represented as

2 $$\overleftrightarrow{T}={\bf{D}}\otimes {\bf{E}}+{\bf{B}}\otimes {\bf{H}}-\frac{1}{2}\overleftrightarrow{I}({\bf{D}}\cdot {\bf{E}}+{\bf{B}}\cdot {\bf{H}}\mathrm{).}$$

The next step is assigning the electric (**E**), magnetic (**H**), electric displacement (**D**), and magnetic induction (**B**) field vectors. The fields were chosen to be a transverse electric wave (electric field polarized perpendicular to direction of propagation)^14,32,33^ of the form:

3 $${{\bf{E}}}_{i}={E}_{0}{e}^{-i{k}_{0}(x\sin (\theta )+z\cos (\theta ))}\hat{{\bf{y}}}$$

4 $${{\bf{H}}}_{i}=\frac{{E}_{0}}{{\eta }_{0}}\,\cos (\theta ){e}^{-i{k}_{0}(z\cos (\theta )-x\sin (\theta ))}\hat{{\bf{x}}}+\frac{{E}_{0}}{{\eta }_{0}}\,\sin (\theta ){e}^{-i{k}_{0}(z\cos (\theta )-x\sin (\theta ))}\hat{{\bf{z}}}$$

5 $${{\bf{D}}}_{i}={\varepsilon }_{0}{{\bf{E}}}_{{\bf{i}}}$$

6 $${{\bf{B}}}_{{\bf{i}}}={\mu }_{0}{{\bf{H}}}_{{\bf{i}}}$$

Inserting the Maxwell stress tensor into the law of conservation of momentum gives us a way to calculate the time-average force exerted on the solar sail. Using the divergence theorem, the gradient of the Maxwell stress tensor can be set equal to the flux of the Maxwell stress tensor through the solar sail surface^23,25,27^.

7 $$\langle F\rangle ={\int }_{V}\nabla \cdot \langle \overleftrightarrow{T}\rangle dV={\oint }_{S}\langle \overleftrightarrow{T}\rangle \cdot \widehat{{\bf{n}}}$$

The Maxwell stress tensor for the incident fields comes out to be

8 $$\overleftrightarrow{{T}_{{\rm{i}}}}=\frac{{E}_{0}^{{\rm{2}}} {\mathcal E} }{{\rm{2}}{\eta }_{0}^{{\rm{2}}}}(\begin{array}{ccc}(\cos ({\rm{2}}\theta ){\mu }_{0}-{\varepsilon }_{0}{\eta }_{0}^{{\rm{2}}}) & 0 & -{\mu }_{0}\,\sin ({\rm{2}}\theta )\\ 0 & ({\varepsilon }_{0}{\eta }_{0}^{{\rm{2}}}-{\mu }_{0}) & 0\\ -{\mu }_{0}\,\sin (2\theta ) & 0 & (\cos (2\theta ){\mu }_{0}-{\varepsilon }_{0}{\eta }_{0}^{{\rm{2}}})\end{array}),$$

where,

9 $$ {\mathcal E} ={{\rm{e}}}^{-{\rm{2i}}(z\cos (\theta )+x\sin (\theta )){k}_{0}}.$$

The derived forces are

10 $$\langle {F}_{i,\perp }\rangle =\frac{{\rm{1}}}{{\rm{2}}}{\varepsilon }_{0}{L}_{i}{L}_{j}{E}_{0}^{{\rm{2}}}\,{\cos }^{{\rm{2}}}(\theta )\widehat{{\bf{z}}}$$

11 $$\langle {F}_{i,||}\rangle =\frac{{\rm{1}}}{{\rm{4}}}{\varepsilon }_{0}{L}_{i}{L}_{j}{E}_{0}^{{\rm{2}}}\,\sin \,(2\theta )\widehat{{\bf{x}}},$$

where *L*_*i*_ and *L*_*j*_ refer to the lengths of the sail edges.

### Using Regular Snell’s Law

The most commonly recognized version of Snell’s law of reflection states that *θ*_*i*_ = *θ*_*r*_. When this is the case, the net force from the incident and reflected waves will have only a normal component. The tangential component of the net force will be negligible because the tangential forces from the incident and reflected waves will be equal and opposite, thus canceling each other out. This is the primary reason why no torque can be generated along the roll axis of the sail. Without any net tangential force along the sail, no rotation can be achieved.

### Using Generalized Snell’s Law

For anomalous reflecting surfaces, *θ*_*i*_ ≠ *θ*_*r*_, so the forces do not cancel out. This means that there is a net tangential force on the sail. Starting from the equation for generalized Snell’s law of reflection, we will derive the forces from anomalously reflected waves. The expression for the generalized Snell’s law of reflection is

12 $$\sin \,{\theta }_{r}-\,\sin \,{\theta }_{i}=\frac{{\rm{1}}}{{n}_{i}{k}_{0}}\frac{\partial {\rm{\Phi }}}{\partial x},$$

which includes a phase gradient, $\frac{\partial {\rm{\Phi }}}{\partial x}$,that is introduced by the metasurface that interacts with the incident light^13–15,22,34–37^.

One particular design that offers considerable promise to maximize the anomalous reflection of incident light is a gradient metasurface. The design consists of an array of symmetric rectangular metal patches with every other patch having a different size in order to induce the appropriate phase gradient for anomalous reflection. These metasurfaces can produce a constant phase gradient that is entirely dependent upon the geometric parameters of the structures.

In the case of the gradient metasurface, the phase gradient will be equal to a constant^14^.

13 $$\frac{{\rm{\partial }}{\rm{\Phi }}}{{\rm{\partial }}{\rm{x}}}=\zeta $$

The dimensions of the phase gradient metasurface can be found by incorporating a modified diffraction equation into the generalized Snell’s law equation, to obtain $\zeta =\frac{2\pi }{{l}_{x}}$, though the length (*l*_*x*_) of the supercell (the metamaterial gradient structure), is not important for our purposes. More generally, we can choose a phase gradient that will provide our desired reflection angle from normally incident light. For example, if we want normally incident light to be reflected at an angle of 90°, then we can use the phase gradient,

14 $$\zeta ={k}_{0}(\sin \,{\rm{90}}^\circ -\,\sin \,0^\circ )={k}_{0}.$$

Once we have chosen a phase gradient, we can then use the value of *ζ* in the generalized Snell’s law expression to solve for the reflection angle

15 $${\theta }_{r}={\sin }^{-{\rm{1}}}(\frac{{\rm{1}}}{{n}_{i}{k}_{0}}\zeta +\,\sin \,{\theta }_{i})\mathrm{.}$$

The plot in Fig. 2b shows how the angle of anomalous reflection differs from the angle of regular reflection. From the plot, it should be mentioned that there is a point where the angle of anomalous reflection becomes imaginary. When $\sin \,{\theta }_{i} > 1-\frac{1}{{n}_{i}{k}_{0}}\zeta$, the value inside of the Arcsine becomes greater than unity, which causes the expression to become complex and the propagating waves are converted into surface waves in the metasurface^38^. The new Maxwell stress tensor is

16 $$\overleftrightarrow{{T}_{r}}=\frac{{E}_{0}^{2}\alpha }{2{k}_{0}^{2}{\eta }_{0}^{2}}(\begin{array}{ccc}-({k}_{0}^{2}{\varepsilon }_{0}{\eta }_{0}^{2}+\beta {\mu }_{0}) & 0 & \delta \\ 0 & {k}_{0}^{2}({\varepsilon }_{0}{\eta }_{0}^{2}-{\mu }_{0}) & 0\\ \delta  & 0 & -({k}_{0}^{2}{\varepsilon }_{0}{\eta }_{0}^{2}-\beta {\mu }_{0})\end{array}),$$

where

17 $$\alpha ={{\rm{e}}}^{({\rm{2i}}(x\zeta +x\sin (\theta ){k}_{0}-z\sqrt{{\rm{1}}-{(\frac{\zeta }{{k}_{0}}+\sin (\theta ))}^{{\rm{2}}}}{k}_{0}))}$$

18 $$\beta =({\rm{2}}{\zeta }^{{\rm{2}}}+{\rm{4sin}}\,(\theta ){k}_{0}\zeta -\,\cos \,(2\theta ){k}_{0}^{{\rm{2}}})$$

19 $$\delta ={\rm{2}}{k}_{0}{\mu }_{0}(\zeta +\,\sin \,(\theta ){k}_{0})\sqrt{{\rm{1}}-{(\frac{\zeta }{{k}_{0}}+\sin (\theta ))}^{{\rm{2}}}}\mathrm{.}$$

The plane wave force was found to be

20 $$\langle {{\bf{F}}}_{{\bf{r}},\perp }\rangle =\frac{{L}_{i}{L}_{j}{E}_{0}^{{\rm{2}}}}{{\rm{4}}{\eta }_{0}^{{\rm{2}}}{k}_{0}^{{\rm{2}}}}({\mu }_{0}(-\,{\rm{2}}{\zeta }^{{\rm{2}}}-{\rm{4}}\zeta {k}_{0}\,sin(\theta )+{k}_{0}^{{\rm{2}}}\,cos({\rm{2}}\theta ))+{\eta }_{0}^{{\rm{2}}}{k}_{0}^{{\rm{2}}}{\varepsilon }_{0})\widehat{{\bf{z}}}$$

21 $$\langle {{\bf{F}}}_{{\bf{r}},||}\rangle =-\,\frac{{\mu }_{0}{L}_{i}{L}_{j}{E}_{0}^{{\rm{2}}}}{{\rm{2}}{\eta }_{0}^{{\rm{2}}}{k}_{0}}(\zeta +{k}_{0}\,sin(\theta ))\sqrt{{\rm{1}}-{(sin(\theta )+\frac{\zeta }{{k}_{0}})}^{{\rm{2}}}}\widehat{{\bf{x}}}$$

Inserting the derived expression for the anomalous angle of reflection into the tangential force equation for *θ*_*r*_, and then adding the result to the incident tangential force, we obtain,

22 $$\langle {{\rm{F}}}_{{\rm{x}}}\rangle ={\langle {{\rm{F}}}_{{\rm{x}}}\rangle }_{{\rm{i}}}+{\langle {{\rm{F}}}_{{\rm{x}}}\rangle }_{{\rm{r}}}=\frac{{\mu }_{0}{L}_{i}{L}_{j}{E}_{0}^{{\rm{2}}}}{{\rm{2}}{\eta }_{0}^{{\rm{2}}}}(\sin (\theta )\,\cos \,(\theta )-\frac{(\zeta +{k}_{0}\,\sin (\theta ))}{{k}_{0}}\sqrt{{\rm{1}}-{(\sin (\theta )+\frac{\zeta }{{k}_{0}})}^{{\rm{2}}}})$$

One thing that is important to note is that the anomalously reflected light should have the same polarization as the incident fields when using the gradient metamaterial structures^14^.

The torque along the roll axis can then be found by crossing each position vector ***r***, from the center point on the sail to a given sail element, with each corresponding differential element of force and adding up all of the contributions^28^. Since there is no y component of force, the torque along the roll axis comes out to be,

23 $$\begin{array}{rcl}\langle {{\rm{\tau }}}_{{\rm{z}}}\rangle  & = & {\int }_{S}\langle d{F}_{y}\rangle x-\langle d{F}_{x}\rangle y=-\,\frac{{\mu }_{0}{E}_{0}^{{\rm{2}}}}{{\rm{2}}{\eta }_{0}^{{\rm{2}}}}(\sin \,(\theta )\,\cos \,(\theta )-\frac{(\zeta +{k}_{0}\,\sin (\theta ))}{{k}_{0}}\\  &  & \times \sqrt{{\rm{1}}-{(\sin (\theta )+\frac{\zeta }{{k}_{0}})}^{{\rm{2}}}}){\int }_{-\,\frac{{L}_{i}}{2}}^{\frac{{L}_{i}}{2}}{\int }_{-\,\frac{{L}_{j}}{2}}^{\frac{{L}_{j}}{2}}ydydx\mathrm{.}\end{array}$$

Since the above integral will be zero if the metasurface is symmetric about the y-axis, it will be necessary to include metasurfaces with different values of *ζ* to break the symmetry. Ideally, metasurfaces with phase gradients that are of equal and opposite signs would be on either side of the y-axis. This could be achieved by a gradient metamaterial structure rotated 180° with respect to its counterpart. If this is the case, the new, optimized torque, *τ*_*z*,*opt*_, can be found to be,

24 $$\begin{array}{rcl}\langle {{\rm{\tau }}}_{z,\mathrm{opt}}\rangle  & = & \frac{{\mu }_{0}{E}_{0}^{{\rm{2}}}{L}_{i}{L}_{j}^{2}}{{\rm{16}}{\eta }_{0}^{{\rm{2}}}{k}_{0}}(({\zeta }_{+}+{k}_{0}\,\sin \,(\theta ))\sqrt{1-{(\frac{{\zeta }_{+}}{{k}_{0}}+\sin (\theta ))}^{2}}\\  &  & -({\zeta }_{-}+{k}_{0}\,\sin \,(\theta ))\sqrt{1-{(\frac{{\zeta }_{-}}{{k}_{0}}+\sin (\theta ))}^{2}}).\end{array}$$

Due to the time-varying nature of the sail system, in future investigations it will be important to investigate dynamically tunable methods to either turn off and on the anomalous reflections, or actively vary the phase gradient of the metasurfaces.

## Results and Discussion

**Figure 3:**
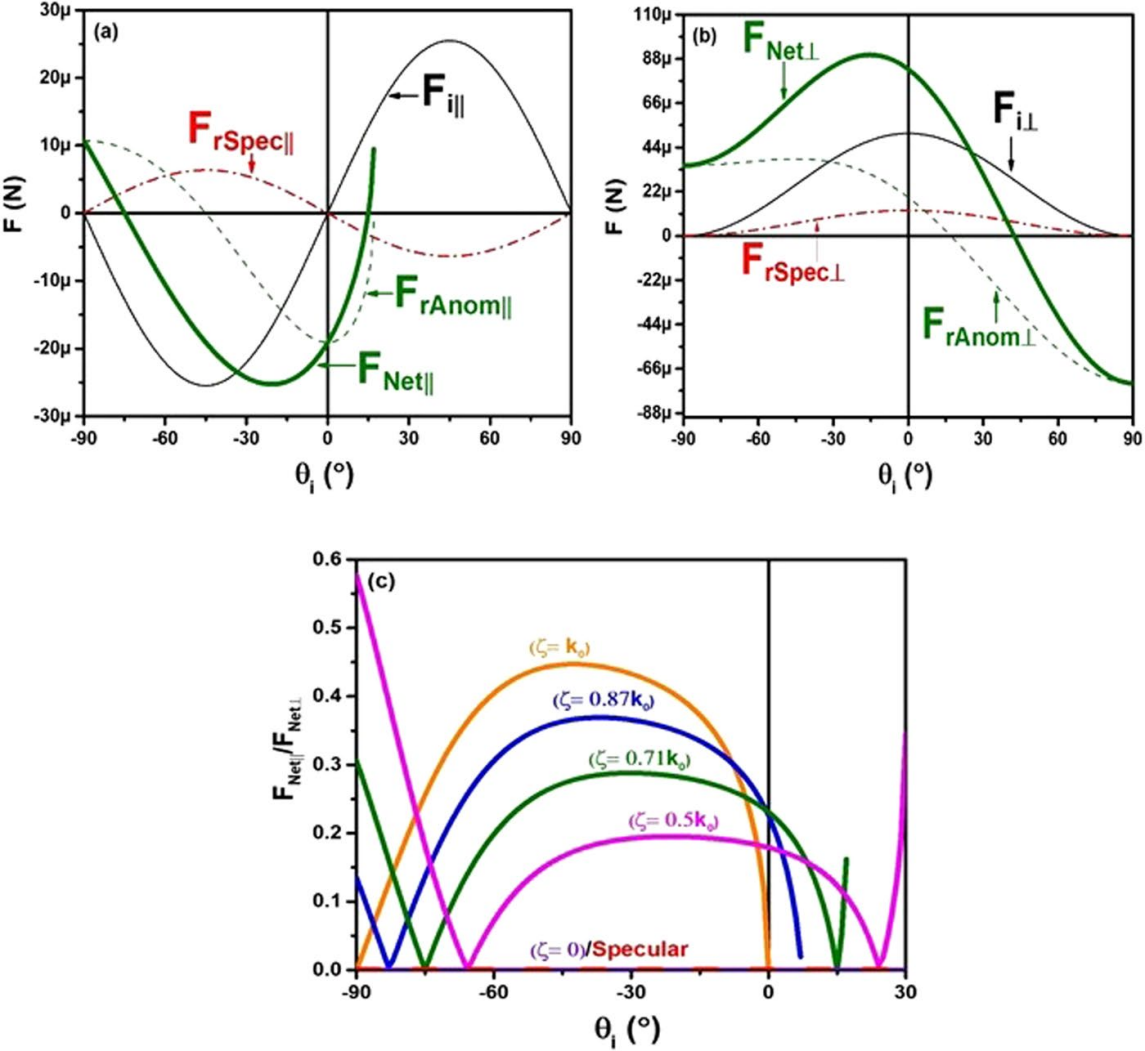
(**a**) Is a plot of the anomalous reflection force (dashed green), specular reflection force (dashed red), incident electromagnetic force (solid black), and net force (bold green) for the lateral components of the solar sail. (**b**) Is the same, but for the normal components. (**c**) Is the ratio of the net forces from solely the specular case (bold red) and from the anomalous cases of varying phase gradient (the rest of the bold colors). All plots have the parameters, *η*_*anom*_ = 0.75. (**a** and **b**) have *ζ* = 0.71*k*_0_.

Figure 3a and b display the force contributions from anomalous reflections and regular reflections in the normal and lateral cases, respectively. In order to make our results consistent with reality, we included an efficiency scaling factor.

The specular and anomalous reflection components are scaled by an anomalous conversion efficiency factor, *η*_*anom*_. It is observed for anomalously reflecting metasurfaces, a certain amount of the reflected light will always be specularly reflected. We chose to use a phase gradient of 0.71*k*_0_, and an anomalous conversion efficiency of 0.75. These values are consistent with^14^. Therefore, the net force on the solar sail can be evaluated by adding the force from the incident wave to the forces from the anomalous and specular reflections, ${\langle {F}_{x}\rangle }_{i}+(1-{\eta }_{anom}){\langle {F}_{x}\rangle }_{r,spec}+({\eta }_{anom}){\langle {F}_{x}\rangle }_{r,anom}={\langle {F}_{x}\rangle }_{Net}$. The bold lines displayed in Fig. 3a and b are the net forces, with the green lines corresponding to the parameters *ζ* = 0.71*k*_0_ and *η*_*anom*_ = 0.75. The plot in Fig. 3c shows the proportion of net lateral force to the net normal force with varying phase gradient *ζ*. All of the colored lines show the ratio of net forces for the cases that include anomalous reflections, $\frac{{\langle {F}_{x}\rangle }_{Net}}{{\langle {F}_{z}\rangle }_{Net}}$, except for the red line, which represents the purely specular case, $\frac{{\langle {F}_{x}\rangle }_{i}+{\langle {F}_{x}\rangle }_{r,spec}}{{\langle {F}_{z}\rangle }_{i}+{\langle {F}_{z}\rangle }_{r,spec}}$, which becomes zero.

In the case of the metasurface we have chosen to focus on (green) in Fig. 3c, the lateral-to-normal force ratio is locally maximized at *θ* = −30° and *θ* = −90°, with the net lateral force being ≈28.8% and ≈30% of the net normal force, respectively. Additionally, there is a consistent ratio for the anomalous case within the range of −45° to −15°, followed by a significant change outside that range. Flat regions on the curve indicate ranges where the angle of incidence will not be a crucial factor to the net force ratios. This is an important aspect to consider in metasurface design for solar sails when choosing a phase gradient that is consistent over a large range of incidence angles. These values will change depending on the anomalous reflection conversion efficiency as well, which will be discussed later.

Nevertheless, an effective way to optimize the lateral-to-normal force ratio of the solar sail is a significant development in metasurface-based attitude control. While rigorous analysis of metasurface placement on the solar sail surface will be carried out in future work, we would like to note that in order for there to be a significant torque along the roll axis of the sail, metasurfaces would ideally be placed a distance away from the center of the sail because this would increase the moment arm, and therefore the torque. In this case, any normal force components would also contribute to a torque along the pitch and yaw axes of the sail as well, which may be undesirable. In order to minimize this effect, it is important to know the optimal angle that produces the largest lateral force in relation to the normal force for a particular metasurface. Our results from Fig. 3c indicate large peaks in this ratio at an incidence angle of −90°, which increase for smaller values of *ζ*. The significance of the results presented can be efficiently realized by designing a metasurface to produce a larger torque along the roll axis in relation to the yaw and pitch axes. However, as *ζ* → 0, the slope becomes steeper, to the point that the incident angle must essentially be exactly −90°, which is parallel incidence and not physically realizable.

**Figure 4:**
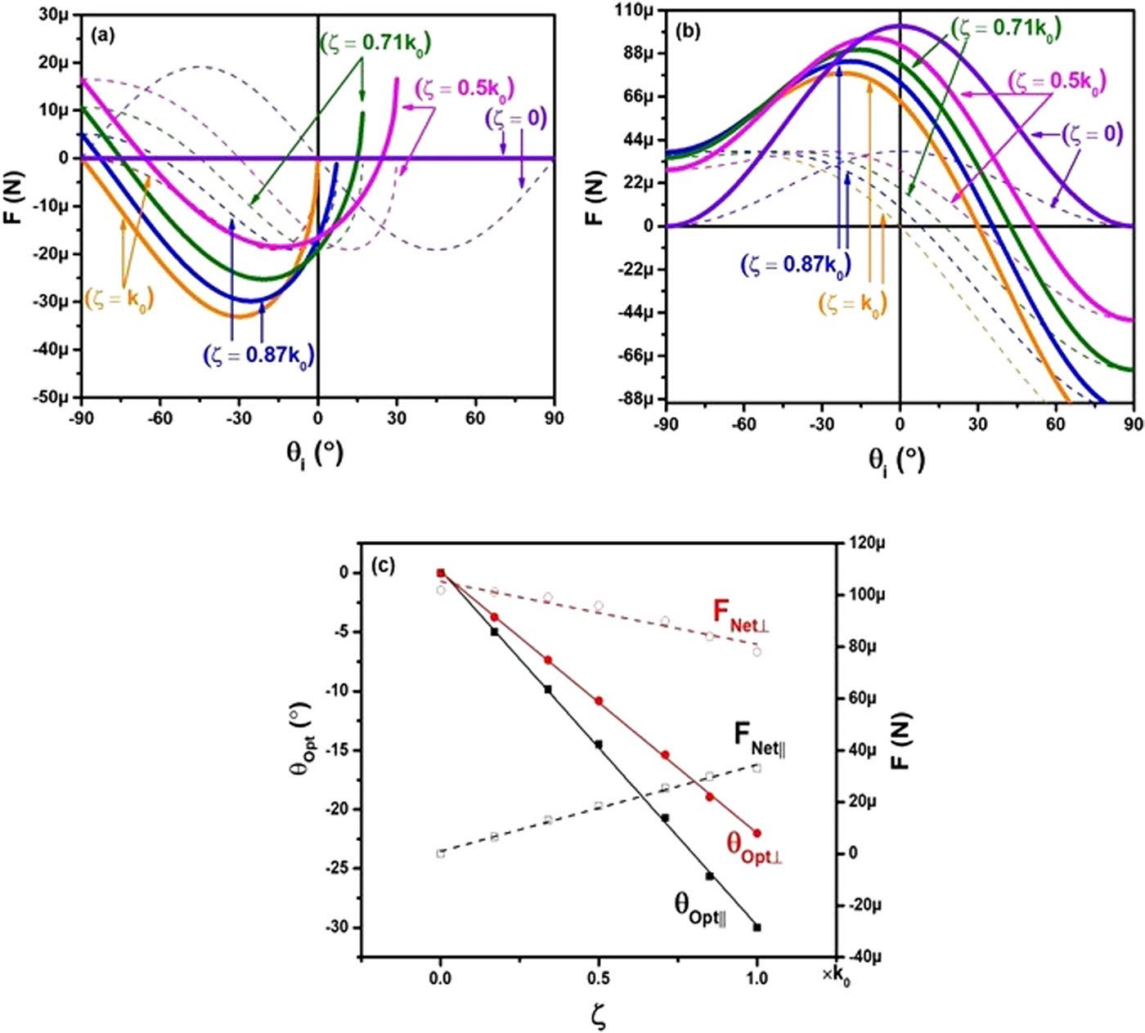
(**a**) Is a plot of the anomalous reflection force (dashed), and net force (bold green) for the lateral components of the solar sail at different phase gradients. (**b**) Is the same, but for the normal components. (**c**) Is the optimal angle that produces the largest magnitude net force for both lateral (black) and normal (red) cases as a function of the phase gradient. All plots have the parameter, *η*_*anom*_ = 0.75.

To further examine the effect a metasurface phase gradient has on the force on a solar sail, Fig. 4 shows the anomalous reflection forces and net forces on the solar sail for different phase gradients. For the lateral forces, as shown in Fig. 4a, a phase gradient of *k*_0_ will create the maximum net lateral force, and a phase gradient of zero will cause the net force to be zero, which is the appropriate result since *ζ* = 0 is equivalent to purely specular reflection. Figure 4b shows the normal forces on the solar sail for comparison. A phase gradient of zero, which corresponds with specular reflection, reaches a maximum net force at normal incidence as expected. As the phase gradient increases, the net normal forces slowly decrease, and with a phase gradient *k*_0_, the lowest net force is produced.

Figure 4c plots the angles that produce the largest net force for each phase gradient and the corresponding forces. The black squares and red circles represent the normal and lateral forces respectively. The solid black lines and symbols correspond to the optimal angles, while the hollow symbols and dashed lines correspond with the optimal forces at these angles. During solar sail missions, it will typically be important for any attitude adjustments to maintain maximum thrust in both lateral and normal trajectories. The angle where both normal and lateral net forces have the same optimal angle correspond only to metasurfaces with *ζ* = 0, and an angle of incidence of 0°. It shows that the larger the phase gradient for the metasurface becomes, the more decoupled the optimal angles for the normal and lateral net forces will be. Therefore, a balance will need to be maintained in order to allow for the greatest possible net lateral force (which occurs when *ζ* is large), and consistency between normal and lateral optimal angles (which occurs when *ζ* is small). This result is particularly important when the desired response is not a net torque, but rather a translational motion of the solar sail system.

**Figure 5:**
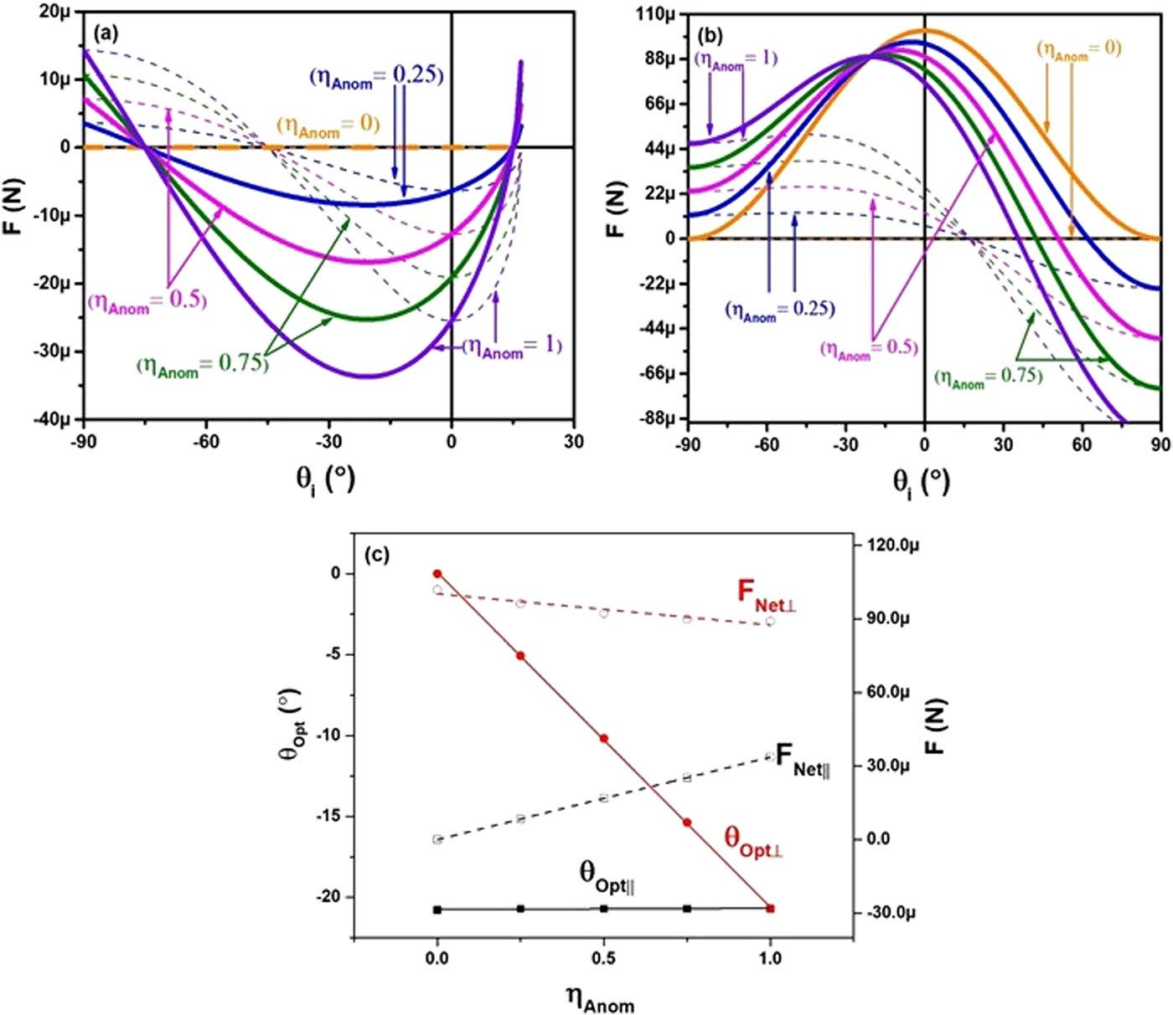
(**a**) Is a plot of the anomalous reflection force (dashed), and net force (bold green) for the lateral components of the solar sail at different values of anomalous conversion efficiency. (**b**) Is the same, but for the normal components. (**c**) Is the optimal angle that produces the largest magnitude net force for both lateral (black) and normal (red) cases as a function of the anomalous conversion efficiency. All plots have the parameter *ζ* = 0.71*k*_0_.

In the final study, we examine the effect of the anomalous conversion efficiency on the forces. Most metasurfaces designed for anomalous reflection have not yet been able to achieve 100% conversion efficiency. A larger anomalous conversion efficiency means that there is more anomalous reflection, which is crucial for producing lateral forces on the sail. Figure 5a supports this claim, as the largest lateral net force occurs when 100% of the incident light is converted into anomalous reflections, and no lateral net force is generated when *η*_*anom*_ = 0. Following suit, Fig. 5b shows that the greatest normal force occurs when the anomalous conversion efficiency is 0%, and the least normal force occurs when it is 100%. Once again, the angles that produce the maximum net forces are examined in Fig. 5c, with respect to the anomalous reflection efficiency. From Fig. 5a and c, it should be noted that for the lateral forces, the maximum net forces are all at the same angle, with only the magnitude of the net force changing with anomalous reflection efficiency. For the normal case in Fig. 5b and c, it can be seen that the optimal angle changes linearly with the anomalous reflection efficiency. The point where both the normal and lateral optimal angles are the same is at an anomalous reflection efficiency of 100%. Once again, this parameter can be taken into consideration when choosing metasurfaces to be further designed and utilized. An ideal case would have *η*_*anom*_ = 1.

## Conclusion

The derivations we have done so far are a vital first step to understanding how metasurfaces could be used with solar sails. We have shown that anomalous reflections from metasurfaces can indeed be used to create tangential forces, and therefore to alter the attitude of a solar sail. Additionally, the metasurfaces would be able to create a net torque along the roll axis of the sail, particularly if they were positioned along the edge of the sail. Metasurfaces can be designed to produce a constant phase gradient that corresponds to the largest magnitude tangential force. From our plots, this phase gradient was found to be *ζ* = *k*_0_. Additionally, we have developed a method to determine the optimum angles to maximize the magnitude of the lateral force on the solar sail in relation to the normal force. For a solar sail system with a metasurface having *η*_*anom*_ = 0.75 and *ζ* = 0.71*k*_0_, there are two optimal angles which produce the largest net lateral-to-normal force ratio. The first angle(≈28.8% of the net normal force) would be at −30°, and the second (≈30% of the net normal force) is at −90°. For decreasing magnitudes of constant phase gradient, the local maximum of the lateral-to-normal ratio increases significantly, though at the expense of approaching a physically unrealizable incidence angle. Finally, we found that for different values of *ζ*, there are ranges of fairly constant lateral-to-normal ratios which can be chosen while picking metasurface phase gradients to use on solar sails. Very small, nonzero, phase gradients have the longest ranges of constant normal-to-lateral ratios, with the added benefit that they have very large maximas approaching *θ* = −90°. This means that they will behave closely to specular reflectors for most incidence angles, and then be able to exhibit very high lateral force at grazing incidences. This work therefore demonstrates the high potential of anomalously reflected light from a metasurface for solar sail control to generate torque along the roll axis of a solar sail.

## Electronic supplementary material

Supplementary Materials
