# Supplementary material for: Strong Solar Radiation Forces from Anomalously Reflecting Metasurfaces for Solar Sail Attitude Control
Source: Sci Rep. 2018 Jul 3;8:10026. doi: 10.1038/s41598-018-28133-2 (PMC6030165; doi:10.1038/s41598-018-28133-2)
Supplement: Supplementary file 1 — Supplementary Materials [file 41598_2018_28133_MOESM1_ESM.pdf]

Strong Solar Radiation Forces from Anomalously Reflecting Metasurfaces for Solar Sail Attitude Control
Supplemental Information

Dylan C. Ullery, Sina Soleymani, Andrew Heaton, Juan Orphee, Les Johnson, Rohan Sood, Patrick Kung, Seongsin M. Kim[1]

[1]Electrical and Computer Engineering, University of Alabama, Tuscaloosa, 35487, United States
[2]NASA, Marshall Space Flight Center, Huntsville, 35811, United States
[3]Aerospace Engineering, University of Alabama, Tuscaloosa, 35487, United States
[*]patkung@eng.ua.edu; seongsin@eng.ua.edu
[+]these authors contributed equally to this work

Supplemental Information

Symbol	Quantity
E_0	Electric Field Amplitude
ε	Electric Permittivity
μ	Magnetic Permeability
ε_0	Free-Space Electric Permittivity
μ_0	Free-Space Magnetic Permeability
η_0	Free-Space Wave Impedance
k_0	Free-Space Wavenumber
n	Refractive Index
θ_i	Angle of Incidence
θ_r	Reflection Angle
Φ	Phase Shift
ζ	Constant Phase Gradient
ζ_+	Positive Valued Constant Phase Gradient
ζ_-	Negative Valued Constant Phase Gradient
η_{anom}	Anomalous Reflection Conversion Efficiency
f	Force Density
E	Electric Field of EM Wave
H	Magnetic Field of EM Wave
D	Electric Displacement Field of EM Wave
B	Magnetic Induction Field of EM Wave
S	Poynting Vector
$\overleftrightarrow{T}$	Maxwell Stress Tensor
$\overleftrightarrow{I}$	Identity Tensor
$L_{i,j}$	Length of Solar Sail Edge
l_x	Length of Metasurface Unit-Cell
P	Periodicity of Unit-Cell
F	Force Vector
r	Position Vector
τ	Torque Vector
τ_{opt}	Optimized Torque Vector From Opposing Metasurfaces

Table 1. List of symbols and parameters used.
